# An Interdisciplinary Approach to Understanding the Psychological Impact of Different Grammaticalizations of the Future

**DOI:** 10.5334/joc.100

**Published:** 2020-05-07

**Authors:** Tiziana Jäggi, Sayaka Sato, Christelle Gillioz, Pascal Mark Gygax

**Affiliations:** 1 University of Fribourg, Department of Psychology, CH; 2 University of Bern, Institute of French Language and Literature, CH

**Keywords:** Grammaticalization of the future, future time reference, reading, sentence processing, time perception

## Abstract

Considering how fundamental and ubiquitous temporal information is in discourse (e.g., Zwaan & Radvansky, 1998), it seems rather surprising that the impact of the grammaticalization of the future on the way we perceive the future has only been scarcely studied. We argue that this may be due to its rather abstract nature and how it has been previously operationalized. In this review, we lay the foundation for studying the impact of the grammaticalization of the future on mental representations of the future by taking an interdisciplinary perspective, connecting cognitive sciences, linguistics, psycholinguistics, economics, and health psychology. More specifically, we argue that experimental psycholinguistics, combined with more applied domains, constitute a promising research avenue.

## Introduction

A growing body of research has demonstrated that grammatical features differing across languages, such as grammatical gender (e.g., Sato et al., 2013; Sera et al., 1994) or motion event construal (e.g., Athanasopoulos & Bylund, 2013a; Flecken et al., 2014), can influence the way in which we mentally represent and perceive the world. However, the role of the future tense in forming mental representations of the world has only scarcely been studied. In this paper, we review the idea that as grammatical features are spontaneously processed, verb tense – the future tense in our case – is a very salient feature to language users, which, in turn, biases their mental representations (e.g., Slobin, 2003). As such, speakers of different languages can experience unconscious shifts of focus and mental representations depending on the emphasized grammatical features used to express the future, even though the information conveyed is held constant (Slobin, 2003).

We argue that the grammaticalization of the future, that is, whether languages use a future tense or not to transmit information about future events (Dahl, 2000), has not received the deserved empirical attention. This neglect seems unusual in light of the importance that thinking about the future has in our daily lives: on average, thoughts about the future occur every 16 minutes (D’Argembeau et al., 2011), and their valence and structure can have important psychological consequences (MacLeod, 2017).

Although research in economics has addressed some level of psychological effect of the grammaticalization of the future, mainly focusing on behavioral economic outcomes (e.g., Chen, 2013), experimental evidence is still scarce, although some authors have started to investigate the effect of the grammaticalization of the future in *temporal discounting* (Chen et al., 2019) and *delayed gratification* (Sutter et al., 2018), both of which will be discussed later. Hereafter, we argue that some experimental paradigms, such as those typical in psycholinguistic research, may constitute a particularly well-suited avenue to address the psychological effects of the grammaticalization of the future.

In this paper, we first review the theoretical and linguistic grounds used to distinguish languages in terms of the way the future is grammaticalized. We then present the mechanisms underlying the construction of mental representations of the future. Finally, we synthesize research on thinking about and referring to the future from different fields to adopt an interdisciplinary framework for future psycholinguistic research on this topic. In highlighting the importance that thinking about the future has in other domains, we offer an overview of the possible impacts grammaticalization could have on the way in which we think about the future. We ground our argument in health-psychological research, which has explored different conceptualizations of thinking about the future. In all, we take an interdisciplinary perspective to motivate future research on how we perceive the future, especially in light of its grammaticalization.

## Linguistic Realizations of the Future

Language provides a set of linguistic features to refer to the future. Within this set, grammatical categories such as verb tense (i.e., location of time of a referred event, e.g., past, present or future tense; Comrie, 1985), aspect (i.e., temporal structure of a referred event, e.g., ongoing or completed event; Comrie, 1976), and modality (i.e., underlying meaning of modal verbs e.g., ability: I *can* go; probability: I *will* go) can be used to mark the future, which is generally referred to as *future time reference* (FTR; Dahl, 2000).

Within a language, FTR varies in that it can be marked in different ways, which range from simple lexical marking, using modal verbs (e.g., in French: *je peux aller à Paris* [I can go to Paris]) or temporal adverbs (e.g., in French: *demain, je vais aller à Paris* [tomorrow, I am going to go to Paris]), to grammatically marked FTR, such as using the future tense incorporated in the verb stem (e.g., in French: *j’irai à Paris* [I will go to Paris]; Bybee, Pagliuca, & Perkins, 1991; Dahl, 2000).

These different ways of marking the future are connected to an immanent feature of talking about the future, which differs from talking about the past or the present: a degree of uncertainty underlying its content. That is, we know the events took place or are taking place in the present and the past, yet we cannot be sure about the future. This degree of uncertainty can translate into expressing intentions (i.e., transmitted with a notion of control over a situation) or predictions (i.e., estimated probability regarding future situations) and is reflected in the choice of FTR (Dahl, 2000). Intentions usually only require lexical marking of FTR and no obligatory grammatical marking (or no future tense; e.g., in French: *la semaine prochaine, je vais à Paris* [next week, I *go* to Paris]), at least within European languages (Dahl, 2000). However, predictions vary much more across languages in their FTR marking (Bybee et al., 1991).

Besides distinguishing between intentions and predictions, temporal context also serves as an indicator of how to mark FTR, especially if a language has several possibilities of grammatically marking FTR. For example, in French one can add a suffix to indicate an inflectional future construction (e.g., *j’ir**ai** à Paris* [I will go to Paris]; composed by a word stem and the suffix -*ai* indicating future tense). Although, depending on the context, one can also use the de-andative periphrastic construction; in other words, *aller* [to go] + infinitive (e.g., *je vais aller à Paris* [I am going to go to Paris]; Dahl, 2000). The temporal context of future events determines which future construction is used. Accordingly, the choice between periphrastic and inflectional constructions depends on how proximal or distant a future event is. Proximal future events are commonly expressed using periphrastic future constructions and are likely to represent intentions (e.g., French: *demain, je vais travailler à la bibliothèque* [tomorrow, I am going to work at the library]). Distant future events are expressed using inflectional future constructions and are likely to represent predictions (e.g., French: *la saison prochaine, la grippe frappera durement* [next season, the flu will hit hard]).

Interestingly, variations in lexical and grammatical marking of FTR can be explained by two different patterns, which evolve diachronically within languages and is referred to as grammaticalization of FTR (Bybee et al., 1991). The first pattern pertains to semantic changes of usual lexical FTR markers through different stages of grammaticalization. Across these stages, the modality of a verb changes as grammaticalization progresses. For example, the original meaning of *shall* was *‘to owe’*, thus derived from an obligational modality; nowadays, *shall* can be used to signal intentional modality (e.g., *I shall get to Paris as soon as I can*). Through grammaticalization of FTR, the meaning of such modal verbs switches from indicating desire, obligation, or ability to signal intention to eventually indicating probability. The second pattern is characterized by the process of formal reduction of the FTR markers over time; in other words, constructions using modal verbs will shorten to morphemes incorporated into the verb stem over time. In French, for example, two such constructions exist at the same time, marking different degrees of grammaticalization (e.g., *je vais aller à Paris* [I am going to go to Paris]; *j’irai à Paris* [I will go to Paris]).

For crosslinguistic comparisons, it is important to consider these stages of grammaticalization (Bybee et al., 1991). Some languages are considered to be in an early stage of grammaticalization, as they only use lexical marking to refer to the future. We consider these languages to have a low degree of FTR (i.e., no future tense) as opposed to languages with grammatical marking of FTR (i.e., some form of inflectional future construction), following Chen (2013). According to the World Atlas of Language Structures (WALS), about 50% of languages have developed inflectional future constructions, meaning a high degree of grammaticalization of FTR (Dahl & Velupillai, 2013). In other languages, FTR expressions often show lower degrees of grammaticalization, such as periphrastic future constructions (e.g., English) or no future tense construction at all (e.g., Indonesian).

Although a formal classification of grammaticalization of FTR constitutes an initial reference point when designing cross-linguistic experiments, different classifications do exist, from dichotomizing FTR (e.g., high vs. low FTR; see Chen, 2013) to defining FTR as a continuous variable (e.g., differentiating between high and low FTR based on topographical features of languages; see Thoma & Tytus, 2018). Importantly, we would argue that these boundaries are not precise, as the classification of some languages have been controversial (see, for example, Radford, 1997 for further discussion on English).

## Considering Cognitive Implications of the Grammaticalization of the Future

### ‘Thinking-for-speaking’ Hypothesis

To address possible effects of FTR grammaticalization on cognition, we argue that Slobin’s (e.g., 2003) ‘thinking-for-speaking’ hypothesis is particularly well adapted. This hypothesis states that as we prepare ourselves to verbally formulate our ideas, we tend to focus on information that is highlighted by the linguistic features specific to a language (e.g., future tense vs. no future tense). Through constant exposure to a given language, speakers’ attention is inevitably directed towards these linguistic features (e.g., present and future are different vs. no linguistic distinction), making them particularly salient. Some have argued that these processes are especially strong with conceptual distinctions that are grammaticalized rather than lexicalized (e.g., Lucy, 1992).

Consequently, FTR grammaticalization may lead to different mental representations of the same future events across speakers of different languages (i.e., languages with different degrees of FTR). Different mental representations of the same future events may even be found within a language (i.e., lexical vs. grammatical marking). This, however, does not necessarily mean that different linguistic features lead to changes in real-world cognition or in general perceptual processes, but that it has an effect on the way we construct, and the content of, mental representations of different events during the processing of linguistic information (Slobin, 2003). Evidence for the ‘thinking-for-speaking’ hypothesis can be found in other domains such as spatial relations (e.g., Bowerman & Choi, 2001), grammatical gender (e.g., Konishi, 1993; Sato et al., 2013) as well as motion event construal (e.g., Athanasopoulos & Bylund, 2013b; Flecken et al., 2014). To our knowledge, no research on linguistic features indicating FTR and their effect on mental representations has been conducted so far.

### Temporal Cognition

Thinking-for-speaking can help us to distinguish cognitive domains that are important to study in order to understand the impact that the grammaticalization of the future has on mental representations of future events. However, we need to draw ideas from existing research on temporal cognition to arrive at possible cognitive conceptualizations of time and of the future to ground our work. A fundamental notion is that time is *spatialized* in language as time metaphors (e.g., Evans, 2003; Núñez & Cooperrider, 2013), meaning that the vocabulary used to talk about time is often borrowed from spatial concepts (e.g., *time passes, time flows* or *the time has arrived*). These time metaphors distinguish between two different ways in which time is considered to move; either with the person themselves (e.g., *the past lies behind me*) or independent of a person’s position (e.g., *June comes after May*). The directionality of these movements varies across cultures (Fuhrman et al., 2011). Research on time found evidence that the temporal information, considered as being abstract, is cognitively organized within concrete spatial concepts (Boroditsky, 2000). Within this spatial conceptualization of time, the future is conceptualized as *lying in front/being ahead of us* (at least in English and French; see, for example, Bylund, Gygax, Samuel, & Athanasopoulos, 2019). This conceptualization not only influences our general conceptualization of time, but can be activated without using language, suggesting a perceptual basis in the representation of time (Casasanto & Boroditsky, 2008).

Although we may assume that the grammaticalization of the future can impact or touch on the perceptual nature of time, the same way time metaphors do, this idea is only speculative at the moment. However, some research on how other temporal information is processed when comprehending text does exist and will be presented next. Although not directly targeting the grammaticalization of the future, we argue that such research offers an interesting platform to study its impact on the representations of time. In the same vein, the following section focuses on future conceptualizations and the role of memory for imagining the future. With these two sections, we aim to introduce a new perspective on the possible cognitive conceptualizations of the future, with a particular focus on its grammaticalization.

### Mental Representations of Time

Although the term mental representation embodies a multitude of concepts, we refer to it as the cognitive construct of life-like representations of the world, such as those initially presented by the seminal works of Johnson-Laird (1983), or Kintsch and van Dijk (1978). While the presentation of the theories and controversies pertaining to mental representations goes beyond the scope of the present paper, it is crucial to understand that mental representations are built through an interaction between the environment (e.g., text, discourse) and the receiver (e.g., reader, listener). To illustrate this, let us consider the sentence *Olivia and George are planning their summer holiday*. In a nutshell, at a surface level of representation, the words and meaning that constitute the sentence are encoded. This level is of special interest for us, as grammatical realizations of the future may be first encoded at this level. We then use this information to build a coherent representation of what the text entails. In this representation, we further add implicit elements that are derived from our general knowledge and that directly interact with the elements already incorporated in our mental representation. For example, since the sentence uses *planning* and mentions *summer*, we can infer in a spontaneous manner that (a) Olivia and George have not yet been on holiday, and (b) It is not *summer* yet. As such, the temporal information *spring* may be encoded, to the extent that readers may even think it was explicit in the sentence.

In fact, temporal information has often been suggested as being part of the dimensions necessary for (text) comprehension (e.g., Zwaan & Radvansky, 1998). Zwaan and Radvansky (1998) stress the ubiquitous nature of temporal information, as even when no temporal cues are mentioned, readers follow the *iconicity assumption*, meaning that they always assume a temporal order (e.g., the narrated order of events is expected to match their chronological order). Others have also discussed the rather central nature of temporal information. In fact, earlier, Zwaan (1996) studied the effect of concretely mentioned temporal cues (e.g., *in an hour*), and showed that reading times increased when processing long time shifts (e.g., *an hour later*) compared with short time shifts (e.g., *a minute later*). Anderson et al. (1983) also showed that readers accessed concepts that were read earlier more efficiently when the shift was congruent with the situation described in the text (e.g., *one hour later at the restaurant*) compared to temporal shifts incongruent with the situation described in the text (e.g., *seven hours later at the restaurant*). Zwaan (1996) confirmed these findings by showing that readers have facilitated access to concepts if they are described within the same time frame as the central situation being depicted.

Other temporal cues, namely grammatical tense and aspect markers, have also attracted some attention in the realm of mental representations (e.g., Becker et al., 2013; Carreiras et al., 1997; Ferretti et al., 2009; Madden & Zwaan, 2003; Magliano & Schleich, 2000). Magliano and Schleich (2000), for example, showed participants narratives that described protagonists in actions that were described either with a perfective or imperfective aspect (i.e., completed vs. incomplete action). When asked about these actions, participants perceived the actions in the imperfective aspect as ongoing and the actions described with the perfective aspect as completed. These results indicated that aspect markers provided processing instructions for the construction of mental representations. Carreiras, Carriedo, Alonso and Fernández (1997) investigated the effect of past and present tense markers on mental representations. Participants read paragraph-long narratives with one critical sentence presented in either the past or present tense. After the critical sentence, a test word was presented either directly, after one filler sentence or after two filler sentences. Participants were asked whether the test word was or was not presented in the preceding text. Results showed that they responded (correctly) more quickly when the test word had been presented in the sentence in the present tense compared to the past tense, suggesting that the information in the present tense was better integrated into participants’ mental representations of the narratives. These results document how temporal information is integrated into mental representations and that different temporal cues can impact mental representations differently, emphasizing the importance of studying the impact of the grammaticalization of FTR in mental representations.

### Perceptual Representation of Time

Mental representations, such as those discussed so far, are not simply constructed via verbal encoding, but their contents may well include perceptual information too. Many have studied such properties, showing that when readers comprehend a text (or discourse more generally), they access perceptual and action-related representations of the described situation (e.g., Barsalou, 1999; Zwaan, 2004; Zwaan & Rapp, 2006). This is true for concrete concepts such as performed actions (e.g., Glenberg & Kaschak, 2002; Zwaan & Taylor, 2006) or an object’s form (e.g., Stanfield & Zwaan, 2001; Zwaan et al., 2002) but also for abstract concepts such as emotions (e.g., Havas et al., 2007), speed (e.g., Fecica & O’Neill, 2010; Speed & Vigliocco, 2014), space (e.g., Dudschig et al., 2012), and most importantly here, time (e.g., Boroditsky, 2000; Casasanto & Boroditsky, 2008). This is not surprising, as those studying metaphors have suggested that seemingly abstract concepts such as time are often translated into concrete ones that have a perceptual basis, such as in *time is a moving object* and *we move through time* (e.g., Lakoff & Johnson, 1980; Casasanto & Boroditsky, 2008).

While research on temporal information in mental representations can help us understand what impact the grammaticalization of the future could have, we still lack information on how the construction of the mental representation is manifested and would thus be conceptualized in an experimental setup. We argue here that research on memory and the future helps further untangle this question and offers some interesting avenues.

### Research on Memory and future Events

Although memory is usually associated with past events, it plays an important role when mentally representing the future. In fact, although the term future is not always presented as such in this domain, concepts such as *prospection*, broadly defined as mentally representing possible future outcomes (e.g., Gilbert & Wilson, 2007), offer us some insight into the possible mechanisms underlying the construction of mental representations of future events. Szpunar et al. (2016), for example, developed a taxonomy of prospection, documenting the many ways one can think of the future. The authors mainly identified four modes of thinking about the future: *simulation* (constructing a comprehensive mental representation of the future), *prediction* (estimating the likelihood of an event in the future), *intention* (determining a goal), and *planning* (organization needed in order to reach a goal). Each mode of thinking about the future is divided into episodic and/or semantic processes. Episodic memory is considered necessary when thinking about a specific autobiographical event in the future (e.g., *I will take some holidays*), semantic memory is relevant for general or abstract future events (e.g., *because of global warming, heat waves will hit us every year*).

Szpunar et al. (2016) highlighted the occurrence of hybrid forms between episodic and semantic memory, which manifest as mental representations based on autobiographical information without a concrete nature (e.g., *I will play an important role in science*). More relevant for our present argument, Szpunar et al. (2016) highlighted the importance of episodic memory, and others have suggested episodic memory to be temporally organized (e.g., Cohn-Sheehy & Ranganath, 2017). Consequently, episodic memory represents events occurring in close succession, at least compared to more distant ones (e.g., Howard & Kahana, 2002; Jenkins & Ranganath, 2010) and people recall items with closer temporal proximity better (e.g., Polyn & Kahana, 2008).

In all, we suggest that different grammaticalizations of the future should lead to different memory structures and that this is grounded in the way future events are represented. For example, it is possible that the grammaticalization of the future can influence whether temporal information is processed as semantic or episodic memory. For example, some abstract information (i.e., semantic memory), such as *because of global warming, heat waves will hit us every year*, could well be encoded as more concrete (i.e., episodic memory) if only weak FTR marking is used. We could further hypothesize that cross-linguistic differences in FTR might impact the way in which such information is (a) processed, (b) retained in terms of memory structure, and (c) subsequently acted upon. We now turn to some indirect empirical evidence to support our perspective.

## From Economy to Health-Psychology: The Explored and the Unexplored

### Linguistic-Savings Hypothesis (LSH)

Previous parts of the paper laid the theoretical foundation to understand relevant processes when thinking about the future as well as the pivotal role grammaticalization of the future may play within these processes. In this section, although we focus on direct applications of this grammaticalization, mainly documented in economic research, we also aim to extend the ideas presented so far to health-psychological domains. Of course, we hope that our considerations will inspire researchers to investigate these concepts more broadly, but we believe that they are particularly relevant for the domains we present next.

In his original paper on the link between FTR strength and the representation of the future, Chen (2013) compared different behavioral indexes, such as saving money, having retirement assets, or adopting healthy behaviors, between countries with different degrees of FTR in their corresponding language. Through his linguistic-savings hypothesis (LSH), he argued that people speaking languages with a higher degree of FTR were less likely to engage in future-oriented behavior. More explicitly, he suggested two possible underlying mechanisms: first, the distance to future events is perceived differently between language groups (i.e., in terms of high vs. low degree of FTR) and second, the concreteness of how future events are perceived between language groups differs. Through greater distance and less concreteness for speakers with a higher degree of FTR, thinking about the future is detached from thinking about the present. To distinguish whether a language was considered to have a high or low degree of FTR, he used a dichotomous criterium (adapted from Dahl, 2000): a low degree of FTR was attributed to languages that do not require the verb tense to mark the future (i.e., weak FTR) in a prediction-based context, whereas a high degree of FTR was attributed to languages that require the verb tense to mark the future (i.e., strong FTR) in a prediction-based context. He found a significant effect of the degree of FTR on behavioral indexes and showed that these effects were independent of other economic or demographic factors (e.g., socio-economic status or origin of the legal system in the corresponding country).

Other studies have shown evidence for the LSH, examining *corporate savings behavior* (Chen et al., 2017), *corporate responsibility* (Liang et al., 2018), and *research and development investment* (Liang et al., 2018; Su et al., 2016). All three domains operationalized the degree of FTR according to Chen’s (2013) dichotomized weak/strong FTR, although Liang et al. (2018) additionally used alternative operationalizations (i.e., inflectional FTR vs. any FTR to code a high degree of FTR). As dependent variables they used variables of different panel data following Chen’s (2003) approach, finding significant negative correlations between FTR and their respective dependent variable.

Further, the LSH was confirmed by research concerning *pro-environmental attitudes* (Kim & Filimonau, 2017), *environmental behavior and policies* (Mavisakalyan et al., 2018) as well as *future-oriented policies in general* (Pérez & Tavits, 2017). Mavisakalyan et al., (2018), for example, followed Chen’s (2003) setup, using panel data from the *World Values Survey*, finding speakers of weak FTR languages to be more willing to engage in costly pro-environmental actions, which was also evident across all countries (e.g., more climate change policies). Kim and Filimonau (2017) assessed pro-environmental attitudes between Mandarin (weak FTR) and Korean (strong FTR) speakers using an online questionnaire. They found significantly higher pro-environmental attitudes in Mandarin speakers suggesting a higher perceived urgency in pro-environmental topics when speaking a weak FTR language (i.e., the future was perceived as being closer). Pérez and Tavits (2017) randomly assigned Russian-Estonian bilinguals to either submit a survey about a “green tax” in Russian or Estonian (strong vs. weak FTR respectively) and found that answering the Estonian questionnaire led to more support for the “green tax”, even after controlling for political conviction.

Although many authors found Chen’s (2013) idea quite promising, others have raised some concerns as to the validity of the LSH. For example, Roberts et al. (2015) argue that Chen’s (2013) results could likely be a statistical artifact from big data analyses, as cultural traits were not factored into the analyses. Hence, they re-analyzed Chen’s (2013) data adding the geographical and historical relatedness of languages as factors and found that the correlations no longer yielded significant results when applying the strictest test of relatedness of languages, suggesting that Chen’s (2013) correlations may have been spurious. Additionally, the operationalization of the dichotomous weak vs. strong FTR can be criticized, given that information from the continuous variable is lost and the threshold between weak vs. strong FTR seems arbitrary from a linguistic point of view (e.g., what would be considered as future tense, periphrastic vs. inflectional forms). Note that this criticism can be generally applied to all studies dichotomizing continuous concepts. Although presenting a detailed account of this issue goes beyond the scope of this paper, we urge researchers to keep such issues in mind.

Roberts et al. (2015) also suggested that experimental paradigms are better suited to investigate the effect of grammaticalization on mental representations of the future, which others have done. Sutter et al. (2018), for example, conducted a study in a bilingual city with primary school children assessing their intertemporal choice preference and compared it between the two language groups (German – weak FTR and Italian – strong FTR). They found that German-speaking children preferred later-larger outcomes significantly more often than their Italian-speaking peers, supporting Chen’s LHS. Chen et al. (2019) conducted an experimental study with Chinese-speaking participants from Singapore and Taiwan. As in Chinese there is no obligatory marked FTR, speakers tend to use two FTR forms, the present tense and a future construction. They set up an intra-linguistic study, presenting participants with the two FTR conditions (using the present tense vs. using the future construction, which can be built according to the English “will + infinitive”-structure) and randomized the conditions in a time preference task. The results did not yield statistical significance but showed a trend in the direction opposite to the LHS. Namely, participants seem to prefer smaller rewards when presented in the future tense and bigger ones when presented in the present tense. It remains unclear whether the trend found constitute a true signal of a possible effect or whether it was only spurious. More experimental research is necessary to clarify these findings.

Interestingly, research investigating the co-evolution of economic and cultural factors found that long-term orientation may have developed as a consequence of pre-industrial crop return (Galor et al., 2016). Long-term orientation hereby refers to a cultural orientation that values future rewards (Hofstede, 1991). Concretely, higher crop return in pre-1500 AD in different geographical regions was linked to higher long-term orientation in those regions, suggesting that a surplus in resources elicited a process of planning for the future. In a subsequent study, Galor et al. (2018) directly linked higher crop return in pre-1500 AD to the existence of periphrastic future tense in languages, finding that periphrastic future tense was more likely to be derived in regions associated with higher crop return in pre-1500 AD and higher long-term orientation. Long-term orientation, however, is not conceptualized as a construct that may easily apply to individuals, as it refers to a value found within cultures (Hofstede, 1991). This means that it cannot be adopted in experimental studies examining the effect of the grammaticalization of the future in mental representations. Hence, similar measures, such as temporal discounting, which will be discussed in the next section, have been proposed.

### Temporal Discounting

*Temporal discounting* and *future time perspective* are concepts that, although primarily used in economic research, have also been applied to health psychology. Both measures address, at an individual level, the way we think about future outcomes (e.g., Teuscher & Mitchell, 2011). Temporal discounting defines a tendency to devalue rewards in the future compared to rewards in the present, even if the rewards are the same, or even larger in the future (e.g., Chapman & Elstein, 1995), whereas future time perspective broadly refers to the sum of a person’s thoughts towards the future within a specific time frame (Lewin, 1951). Both concepts have been widely studied indicating correlations with various health related outcomes such as well-being, substance use and physical exercise (e.g., future time perspective: Kooij et al., 2018; temporal discounting: Story et al., 2014). Namely, those preferring later rewards can be characterized as driven by long-term outcomes. In fact, temporal discounting has even been proposed as a potential behavioral marker for addiction (Bickel et al., 2014), as those suffering from diverse addictions have been shown to prefer short-term over long-term rewards than those without any addiction (Bickel et al., 2007), except for coffee addiction (Jarmolowicz et al., 2015). Thoma and Tytus (2018) adapted the temporal discounting paradigm to investigate the effect of the grammaticalization of the future on perceiving future outcomes. Unlike previous studies, they operationalized FTR strength as a continuous variable and examined five languages ranging from a low degree of FTR to a high degree of FTR (in increasing FTR order: Chinese, German, Danish, Spanish, English). Participants read ten decision scenarios and had to choose either sooner-smaller or later-larger economic and health-related outcomes (e.g., *Your doctor recommends to eat more healthily in order to lower your BMI*: *ignore advice, enjoy good food and live now* or *take advice, change diet and lower your BMI for the next visit*). Across all languages, they found a bias towards the later-larger condition; against their expectations, speakers of a high degree of FTR languages showed a bigger bias for later-larger choices. The authors argue that this result is due to the hypothetical nature of the decision scenarios leading participants to answer in a more biased manner. In all, although these studies represent promising avenues of research, their results are not yet conclusive as to the impact of future grammaticalization on the way we perceive the future.

### Mental Time Travel

Another line of research in the health-psychological context has focused on mental time travel, which refers to imagining one’s own future as vividly as possible (e.g., MacLeod, 2017), yet it has never addressed the possibility of focusing on different grammaticalizations of the future. The concept of mental time travel has been studied regarding well-being, depression, and anxiety, especially focusing on patterns of positive and negative thoughts related to the future (e.g., MacLeod & O’Conner, 2018). For example, in healthy controls, positive thoughts about the future are related to positive affect, but not negative affect. Vice versa, negative thoughts about the future are associated with negative affect, but not positive affect. Compared with healthy controls, in clinically depressed patients, positive thoughts about the future are reduced, whereas the amount of negative thoughts about the future remains unchanged (MacLeod & O’Conner, 2018). Research combining mental time travel and the grammaticalization of the future may be eventually applied when developing interventions to reduce depressive symptoms. More specifically, given that the mental processes discussed so far may prove to be relevant, verb tense (or other markers for that matter) may well trigger different processes depending on the temporal distance they imply. This could be of relevance either for multilingual or for monolingual patients within their own language. For the latter patients, this will depend on the very existence of different FTRs within the language, and possible effects may well be explained in terms of *framing effects* (as discussed by Gross (1998) in relation to emotion regulation). For the former group, the issues at stake are reminiscent of those discussed by Monaco et al. (2019) on the clinical effects linked to embodied cognition in L2 (i.e., non-native language). Namely, many clinical professionals deal with migrant populations compelled to interact in L2. Research on the impact of these interactions, in terms of embodiment (e.g., Monaco et al., 2019), or in terms of different grammaticalization, on clinical outcomes are still scarce.

### Big Data Approach

Evidence that thinking about the future has an influence in health-psychological contexts derives from big data using Twitter. For example, Thorstad and Wolff (2018) analyzed the content of over 90 million tweets and found that future-sightedness (i.e., how far into the future people think) was related to decision-making. Far future-sighted people connected the future more closely to the present compared to close future-sighted people, indicating more blurred distinctions between present and future representations. This is highly reminiscent of differences between strong and weak FTR. Note that in terms of operationalizing future-sightedness in tweets, the authors used a SUTime tagger measure, which tags regular expression patterns with a combination of keywords and rules to recognize temporal expressions. Hence, the measure did not directly assess grammatically or lexically marked FTR. A similar paper studied tweets regarding their future orientation (i.e., frequency of referencing the future) in different counties of the USA and their association of HIV prevalence within the county (Ireland et al., 2015). Interestingly, the authors found that future-oriented language in tweets buffered health risk, in other words, referencing the future was associated with lower HIV prevalence. Future orientation in tweets was assessed using the text analysis tool LIWC2007, including a category ‘future tense’ (using mainly modal verbs such as *will* or *should* to detect FTR; Tausczik & Pennebaker, 2010). Importantly, and maybe unfortunately for the questions at stake in our paper, this category was removed in the later version of the LIWC due to poor psychometric criteria and replaced by the category ‘future focus’ (Pennebaker et al., 2015).

In all, these different results speak to a link between certain language constructions and one’s orientation towards the future. So far, big data analyses could be used as a hypothesis-generating tool regarding the grammaticalization of FTR and its presumed effect on health-psychological cognition. As such, future research in that direction might benefit from big text data analyses yet incorporating different degrees of grammaticalization and further testing their effects in experimental paradigms.

## Conclusions and Propositions for Future Research

Studying the grammaticalization of the future with a thinking-for-speaking approach seems a viable research avenue, as linguistic literature suggests that different degrees of FTR exist within languages but also across languages. Usually, languages with higher degrees of FTR additionally use particular constructions with lower degrees of FTR, as seen in the French inflectional and periphrastic constructions, to express a range of probability regarding future events (Bybee et al., 1991). There are no clear criteria to identify languages with a high degree of FTR: some studies categorize periphrastic future constructions as a high degree of FTR (e.g., Dahl & Velupillai, 2013; Galor et al., 2018), whereas other studies use the general marking of the verb tense to characterize a high degree of FTR (e.g., Chen, 2013; Kim & Filimonau, 2017; Mavisakalyan et al., 2018). Future research may consider grammaticalization of the future as a continuum (e.g., Thoma & Tytus, 2018) and adapt the categorization of the degree of FTR to suit a specific research question.

Psycholinguistic research has clearly identified the importance of time as a necessary dimension to construct coherent mental representations of text and discourse. Following Slobin’s (e.g., 2003) ‘thinking-for-speaking’ hypothesis, we suggest that the grammaticalization of the future may impact mental representations of future events, as it alters the way it implicitly conveys information about uncertainty and distance of future events. Research on these issues have been scarce, which may be due to the difficulty to (a) operationalize grammaticalization of the future and (b) operationalize the representation of the future. We hope that we have provided solutions to these issues, mainly to motivate research along these avenues. We believe that these definitional issues constitute fascinating challenges for research. Possible operationalizations of the representation of the future could be based on Chen’s (2013) LSH, where he proposes differences in perceived *concreteness* or *certainty* and distance between language groups. Distance as an alternative operationalization would follow the linear space-time relation found in research of time metaphors (Boroditsky, 2001), where time is conceptualized as a timeline suggesting that the future is simply spatially further away compared to the present.

Research in economics has already applied the grammaticalization of the future to research questions comparing differences in *saving behaviors, retirement assets*, and *smoking behaviors* across countries speaking different languages (Chen, 2013; Liang et al., 2018), yet their observations have also been questioned. Although their results indicated some level of correlation between degrees of FTR and various behavioral economic outcomes, their methodological approaches may have been prone to the overestimation of these correlations (Roberts et al., 2015). Consequently, we would join Roberts et al.’s (2015) observation that experimental work may be better suited to address these issues.

Finally, if the link between grammaticalization of the future and the way we perceive the future may prove to be a viable scientific avenue, it may be applied to health psychology. Indeed, literature seems to indicate that thinking about the future is a central concern in this domain (Teuscher & Mitchell, 2011). For example, research suggests that thinking about the future influences our well-being and mental health (MacLeod & O’Conner, 2018). The relevance of thinking about the future in health psychology combined with the theoretical implications we have discussed regarding the grammaticalization of the future compose a synthesis that could lead to exciting new research avenues. In general, the challenge for applied research using psycholinguistic approaches will be to find suitable behavioral outcomes. Future interdisciplinary research should be aware of the considerations offered in this review.
